# Dopamine D2-Subtype Receptors Outside the Blood-Brain Barrier Mediate Enhancement of Mesolimbic Dopamine Release and Conditioned Place Preference by Intravenous Dopamine

**DOI:** 10.3389/fncel.2022.944243

**Published:** 2022-07-12

**Authors:** J. Daniel Obray, Christina A. Small, Emily K. Baldwin, Eun Young Jang, Jin Gyeom Lee, Chae Ha Yang, Jordan T. Yorgason, Scott C. Steffensen

**Affiliations:** ^1^Department of Psychology and Neuroscience, Brigham Young University, Provo, UT, United States; ^2^Research Center for Convergence Toxicology, Korea Institute of Toxicology, Daejeon, South Korea; ^3^College of Korean Medicine, Daegu Haany University, Daegu, South Korea

**Keywords:** dopamine, microdialysis, dopamine 2 receptor, domperidone, ventral tegmental area, nucleus accumbens, mesolimbic pathway

## Abstract

Dopamine (DA) is a cell-signaling molecule that does not readily cross the blood-brain barrier. Despite this, peripherally administered DA enhances DA levels in the nucleus accumbens and alters DA-related behaviors. This study was designed to investigate whether DA subtype-2 receptors are involved in the enhancement of nucleus accumbens (NAc) DA levels elicited by intravenous DA administration. This was accomplished by using microdialysis in the NAc and extracellular single unit recordings of putative DA neurons in the ventral tegmental area (VTA). Additionally, the reinforcing properties of intravenous DA were investigated using a place conditioning paradigm and the effects of intravenous DA on ultrasonic vocalizations were assessed. Following administration of intravenous dopamine, the firing rate of putative DA neurons in the VTA displayed a biphasic response and DA levels in the nucleus accumbens were enhanced. Pretreatment with domperidone, a peripheral-only DA D2 receptor (D2R) antagonist, reduced intravenous DA mediated increases in VTA DA neuron activity and NAc DA levels. Pretreatment with phentolamine, a peripheral α-adrenergic receptor antagonist, did not alter the effects of IV DA on mesolimbic DA neurotransmission. These results provide evidence for peripheral D2R mediation of the effects of intravenous DA on mesolimbic DA signaling.

## Introduction

Dopamine (DA) is a cell-signaling molecule. In addition to being synthesized within the major DAergic pathways inside the brain, DA is also synthesized outside the brain. Peripheral DA comes from a number of sources including the gastrointestinal tract (Eisenhofer et al., 1997), the sympathetic ganglia and adrenal medulla (Shepherd and West, 1953; Libet and Tosaka, 1970; Kokubun et al., 2019), and leukocytes (Josefsson et al., 1996; Cosentino et al., 2002; Laukova et al., 2013; Gopinath et al., 2020). In the brain, DA plays a key role in processes such as motor coordination (Emerich et al., 1993; Avila-Luna et al., 2018), locomotor activity (Iversen, 1970; Gatica et al., 2020), saliency (Cieslak and Rodriguez Parkitna, 2019; McCutcheon et al., 2019), and reinforcement learning (Schultz et al., 1993; Dabney et al., 2020; Lak et al., 2020). In the periphery, DA is involved in processes such as the regulation of gut motility (Schuurkes and Van Nueten, 1984; Auteri et al., 2016), renal function (McDonald et al., 1964; Meyer et al., 1967), blood pressure (Russell, 2019), and immunity (Ambrosi et al., 2017; Kawano et al., 2018).

Dopamine does not readily cross the blood-brain barrier (BBB), although following intranasal and, to a lesser degree, intravenous (IV) administration DA enters neurons of the olfactory tract (Dahlin et al., 2000, 2001). This may result from uptake by DA transporters in the nasal mucosa (Chemuturi et al., 2006). Intranasal DA enhances DA release in the nucleus accumbens (NAc; de Souza Silva et al., 2008), modulates locomotor activity (Buddenberg et al., 2008; de Souza Silva et al., 2016), and enhances performance on memory tasks (Trossbach et al., 2014; Wang et al., 2017). Further, intranasal DA has been shown to decrease hyperexcitability, improve attention, and ameliorate deficits in reversal learning (Ruocco et al., 2009, 2014; Li et al., 2021). Negative affect related vocalizations in response to restraint are also decreased by intranasal DA (Talbot et al., 2017).

Dopamine receptors are grouped into two classes, dopamine 1-like receptors (D1Rs) and dopamine 2-like receptors (D2Rs) based on their pharmacological and structural characteristics, as well as differences in their modulation of cAMP (Kebabian, 1978; Spano et al., 1978; Civelli et al., 1993). Dopamine receptors in the brain are expressed in many regions including the striatum, olfactory tubercle, substantia nigra (SN), ventral tegmental area (VTA), hypothalamus, and nucleus of the solitary tract (NTS; Bouthenet et al., 1991; Lawrence et al., 1995; Hyde et al., 1996; Le Moine and Bloch, 1996). In the periphery, DA receptors are expressed in sympathetic ganglia, adrenal glands, the heart, the kidneys, blood vessels, the gastrointestinal tract, and immune cells (Missale et al., 1998; McKenna et al., 2002).

Recent research has highlighted vagally mediated signaling pathways that modify VTA DA neuron activity and related behavior (Han et al., 2018; Fernandes et al., 2020; Brougher et al., 2021). Dopamine 2 receptors are expressed within these pathways, and play an important role in modulating neurotransmission, particularly within the NTS (Kline et al., 2002). This study was designed to investigate the possible role of peripheral D2Rs in mediating some of the effects of peripherally administered DA.

We recently demonstrated that the peripheral-only D2R antagonist domperidone (DOM) attenuated ethanol’s enhancement of mesolimbic DA release and decreased ethanol-induced sedation, suggesting that peripheral D2Rs mediate some of the effects of ethanol on brain DA release (Obray et al., 2022). While previous experiments have provided indirect evidence of enhanced neuronal DA release following peripheral administration of DA this has not been directly tested (Pum et al., 2009). It was hypothesized that peripherally administered DA would, at least in part, enhance DA levels through a peripheral D2R-dependent mechanism. It was also hypothesized that this enhancement would be neuronally mediated. Finally, it was anticipated that IV DA would be modestly reinforcing in a place conditioning paradigm. These hypotheses were tested by recording from putative DA neurons in the VTA and by measuring NAc DA levels following IV DA while blocking action potential-dependent DA release. Finally, IV DA was evaluated for reinforcing properties using a place conditioning paradigm.

## Materials and Methods

### Animal Subjects

Male Wistar rats weighing more than 300 g from a breeding colony at Brigham Young University (BYU) were used for these studies (Table 1). All rats were socially housed in a temperature and humidity-controlled environment. Subjects were given *ad libitum* access to food and water. The lighting in the room where subjects were housed was maintained on a 12 h/12 h light/dark schedule for the duration of the experiments. All experimental procedures were approved by the Institutional Animal Care and Use Committee at Brigham Young University and Daegu Haany University and carried out in accordance with NIH guidelines.

**Table 1:** This table contains information about the number of animals used in the experiments as well as any important notes about their use.

Experiment	Number of animals	Notes
Microdialysis: IV DA	26	No more than three doses of IV DA/animal (separate sessions)
Microdialysis: DOM dose response	5	
Microdialysis: Drug effects	8	Each rat received VEH, PHENT, ETIC, DOM (separate sessions)
Microdialysis: Modified aCSF	11	Two doses of IV DA/animal (same session)
Single-Unit	26	Nine animals (VEH, DOM) Eight animals (PHENT) One dose of IV DA/animal
Place Conditioning	60	10 animals/group Locomotor and USV data also collected
Total	136	

### Drugs and Chemicals

Dopamine hydrochloride (H8502, Sigma-Aldrich, Saint Louis, MO, USA), eticlopride hydrochloride (E101, Sigma-Aldrich), quinpirole hydrochloride (1061, Tocris Bioscience, Bristol, UK), and phentolamine hydrochloride (P7547, Sigma Aldrich) were all prepared fresh prior to each injection by dissolving in 0.9% NaCl. Domperidone (J63681, Alfa Aesar, Haverhill, MA, USA) was mixed in a solution of 0.02% acetic acid and gently heated until fully dissolved. Heparin sodium (American Pharmaceutical Partners, Incorporated, Schaumberg, IL, USA) was diluted to 10 units per milliliter in 0.9% NaCl. Gentamicin sulfate (510209, VetOne, Boise, ID, USA) was diluted to 5 mg/ml in 0.9% NaCl. The injection volume for each of these drugs was 1 ml/kg. Each of these drugs was administered IV. Urethane (44804-30, Alfa Aesar) was dissolved at a concentration of 150 mg/ml in 0.9% NaCl and administered at 8 ml/kg intraperitoneal (IP). All procedures using urethane anesthesia were terminal.

### Surgical Procedure

For IV drug administration, rats were implanted with a catheter in the jugular vein. The catheter was passed subcutaneously to exit the back of the rat through an infusion guide cannula (313-000BM-10-5UP/SPC, Plastics One, Roanoke, VA, USA). At the end of the surgery, the wound was irrigated with gentamicin sulfate. After implantation, patency was maintained by a daily flush of heparinized saline. For microdialysis, a guide cannula (MD-2250, Bioanalytical Systems Incorporated (BASI), West Lafayette, IN, USA) was implanted in the nucleus accumbens (NAc) at the following coordinates from bregma: +1.7 mm anteroposterior (AP), +0.8 mm mediolateral (ML), and −6.0 mm dorsoventral (DV). All subjects were anesthetized for the duration of the procedure using isoflurane (1.5%–2.0%). Subjects were given one week to recover post-surgery and received post-operative care including buprenorphine (0.5–1.0 mg/kg, IP) twice daily for 2 days post-surgery, and carprofen (2 mg) in an edible tablet once a day for 1-week post-surgery.

### Microdialysis and High-Performance Liquid Chromatography

On the test day, microdialysis probes (MD-2200, BASI) were inserted into the NAc through the guide cannula while the subject was briefly anesthetized with isoflurane (4%). Artificial cerebral spinal fluid (aCSF) containing 148 mM NaCl, 2.7 mM KCl, 1.2 mM CaCl_2_, and 0.85 MgCl_2_ (pH = 7.4) was perfused through the probe at a rate of 2.0 μl/min. During experiments testing for physiological release of DA a modified aCSF containing no calcium and 37 mM lidocaine was perfused (Westerink et al., 1987; Herrera-Marschitz et al., 1992). Samples were collected over several hours and analyzed every 20 min until a stable baseline lasting at least 2 h was established. After obtaining a stable baseline, DOM (1 mg/kg), phentolamine (PHENT; 1 mg/kg), or vehicle (VEH; 1 ml/kg) was injected 10 min prior to injection with either DA (0.1–3.0 mg/kg) or VEH (1 ml/kg). The range of doses for IV DA were selected based on literature indicating that doses of 3.0 mg/kg (intranasal) and 30 mg/kg (IP) were required to enhance NAc DA levels (de Souza Silva et al., 2008). Dialysate samples were then collected and analyzed for an additional 2 h. Determination of the DA content in the dialysate was performed using high-performance liquid chromatography (HPLC) pump (Ultimate 3000, Dionex) connected to a coulometric detector (Coulochem III, ESA). The coulometric detector included a guard cell (5020, ESA) set at +275 mV, a screen electrode (5014B, ESA) set at −100 mV, and a detection electrode (5014B, ESA) set at +220 mV. DA was separated using a C18 reverse phase column (HR-80, Thermo Fisher Scientific, Waltham, MA, USA). Mobile phase containing 90 mM NaH_2_PO_4_, 50 mM citric acid, 1.7 mM 1-octanesulfonic acid, 50 μM ethylenediaminetetraacetic acid, 10% v/v acetonitrile, and 0.3% v/v triethylamine (final pH = 3.0 by phosphoric acid) was pumped through the system at a flow rate of 0.5 ml/min. The detection limit for DA was 600 pM. External DA standards (1 nM, 10 nM, and 100 nM) were assayed concurrently with the samples to allow for the construction of a calibration curve using Chromeleon (Thermo Fisher Scientific) which was then used to estimate the DA concentration of the dialysate samples. DA levels following drug administration are expressed as a percentage of the baseline DA levels. Baseline DA levels are computed as the average DA concentration of a minimum of three consecutive stable collections occurring prior to drug administration (defined as three consecutive collections within 10% of the moving average of the previous three collections). The average baseline DA concentration was 7.8 ± 0.7 nM.

### Domperidone Dose Response Measurement

The effect of increasing concentrations of DOM (0.005 mg/kg–1.0 mg/kg) on IV DA (1.0 mg/kg) enhancement of NAc DA levels was assessed. Sixty minutes after achieving a stable baseline, PHENT (1 mg/kg) was injected 10 min before DA (1 mg/kg). After another 60 min had elapsed PHENT (1 mg/kg) + DOM (0.005 mg/kg) was administered 10 min before IV DA (1 mg/kg) was given. After another 60 min PHENT (1 mg/kg) + DOM (0.01 mg/kg) was injected 10 min before the next administration of IV DA. This pattern continued for DOM (0.1 mg/kg), (0.5 mg/kg), and (1.0 mg/kg) with 60 min separating each injection. Brain dialysates were collected every 10 min for the duration of the experiment and were analyzed for DA concentration as described above.

### Recording of VTA DA Neuron Spikes

For the extracellular recording of VTA DA neuron spikes, rats were anesthetized using urethane (1.2 g/kg, ip). An IV catheter was then implanted, and a micropipette electrode was inserted into the VTA using an Inchworm 8200 microdrive (EXFO Burleigh) at the following coordinates from bregma: −5.3 AP, +0.6 to +1.0 mm ML, and −7.8 to −9.2 mm AP to −5.7 AP, +0.4 to +0.6 ML, and −8.0 to −8.4 DV. Body temperature was maintained at 37.4 ± 0.2°C for the duration of the surgery using a feedback-regulated heating pad. Once a suitable neuron had been identified, baseline activity was recorded for a minimum of 5 min after which PHENT (1 mg/kg), DOM (1 mg/kg), or VEH (1 ml/kg) was administered, and the firing rate of the neuron was recorded for an additional 5 min. After these 5 min had elapsed, DA (3 mg/kg) was administered, and the neuron was recorded for another 20 min at which point quinpirole (0.1 mg/kg) was administered. No more than one neuron was recorded per animal. The average firing rate was computed for the baseline period, the pretreatment period, the first 90 s (average duration of the inhibition) following IV DA administration, and 330 s (average duration of the excitation) centered on the excitation phase. The firing rate for each period was then expressed as a percentage of the baseline firing rate and analyzed. The average baseline firing rate of all neurons was 3.7 ± 0.5 events/s (Table 2).

**Table 2:** This table contains information about the firing rate of recorded neurons.

Treatment group	Baseline firing rate (mean ± sem)	Baseline firing rate range (Hz)
VEH + IV DA	3.5 ± 0.9	0.6–8.7
PHENT + IV DA	3.7 ± 0.8	1.2–8.4
DOM + IV DA	3.9 ± 0.9	0.9–8.8

Extracellular potentials were recorded using 3.0 M KCl filled micropipettes (4–10 MΩ). Potentials were amplified by a multiclamp 700 A amplifier (Axon Instruments). Single-unit activity was filtered at 300 Hz to 10 KHz for “filtered” recordings and filtered at 0.1 Hz to 10 KHz for “unfiltered” recordings and displayed on a digital oscilloscope (TDS 2002, Tektronix, Beaverton, OR, USA). Potentials were sampled at 20 KHz with National Instruments (Austin, TX, USA) data acquisition boards in Macintosh computers (Apple, Cupertino, CA, USA). Recorded action potentials were discriminated using a WP-121 spike discriminator [World Precision Instruments (WPI), Sarasota, FL, USA]. Discriminated spikes were captured by a National Instruments NB-MIO-16 digital I/O and counter timer acquisition board.

VTA DA neurons were identified using previously established criteria (Ungless and Grace, 2012). These criteria included: a relatively slow baseline firing rate (0.5–12 events/s), a biphasic positive-negative action potential, and a start to trough action potential duration of equal to or greater than 1.1 ms during “filtered” recordings (Ungless et al., 2004), and strong inhibition (>90%) of firing rate by systemic administration of quinpirole (0.1 mg/kg).

### Place Conditioning and Locomotor Activity

The place conditioning apparatus consisted of a 32" × 16" × 16" plexiglass box subdivided into two 16" × 16" × 16" compartments by a guillotine door. The apparatus was housed inside a sound-attenuating cubicle. The two compartments were distinguished by a rough acrylic floor inside one compartment and a smooth plexiglass floor inside the other. Both floors were suspended off the base of the apparatus by 1" with a piezoelectric sensor mounted in the center of the floor. The signal from the piezoelectric transducer was amplified (10×) and filtered (0.1–100 Hz) using a CyberAmp 820 amplifier (Axon Instruments). This signal was then digitized at 100 samples/s using a National Instruments board on a PXI-1011 chassis connected to a Windows-PC (Microsoft, Redmond, WA, USA). Recorded piezoelectric signals were analyzed for locomotor activity by calculating the root mean square (RMS) using Igor Pro 7 (Wavemetrics, Incorporated, Lake Oswego, OR, USA). Additionally, piezoelectric signals were analyzed using custom software to determine how much time each rat spent in each chamber. We have found this apparatus to be biased for Wistar rats with Wistar rats showing a moderate initial preference for the smooth floored compartment.

The place conditioning paradigm consisted of three phases: pretest, conditioning, and posttest. In the pretest phase, the guillotine door was removed, and the rats were allowed 30 min to explore the apparatus. Initial preference and locomotor activity were recorded for each rat. The conditioning phase consisted of four trials pairing VEH (1.0 ml/kg) + DA (0.1 or3.0 mg/kg), DOM (1.0 mg/kg) + DA (0.1 or 3.0 mg/kg), DOM (1.0 mg/kg), or VEH (1.0 ml/kg) with the less preferred chamber and four trials pairing saline (1.0 ml/kg) with the more preferred chamber. These trials lasted for 10 min each and the rats received two trials per day for 4 days. Saline pairing trials were completed in the morning while DA (or equivalent) pairing trials were completed in the evening. Locomotor activity was recorded for each rat during each conditioning trial. Finally, the posttest phase was identical to the pretest phase and occurred on the day after the final conditioning trial. A difference score was computed for each rat using the time spent in the CS+ chamber at the pretest and posttest (difference = posttest − pretest). The units of the posttest score were expressed in standardized units of s/min. Conditioned preference and locomotor activity were recorded for each rat.

### Ultrasonic Vocalizations

Ultrasonic vocalizations (USVs) were recorded during each conditioning trial for each rat. Recordings were performed using a miniMIC ultrasonic microphone (Binary Acoustic Technology, Tucson, AZ, USA) attached to a Windows PC (Microsoft) running SCAN’R software (Binary Acoustic Technology). The recorded files were then analyzed for USVs using DeepSqueak (Coffey et al., 2019). After the initial analysis using DeepSqueak the files were then reviewed for false positives by a technician who was blind to the experimental conditions. Ultrasonic vocalizations were classified as being negative affect related (18 KHz–32 KHz; Knutson et al., 2002; Kagawa et al., 2017) or positive affect related (32 KHz–96 KHz; Knutson et al., 1998, 2002; Scardochio et al., 2015), and the total number of vocalizations was summed across the four CS+ sessions for each rat and used in the subsequent statistical analysis.

### Statistical Analysis

Statistical analyses were performed using Stata 15.1 (StataCorp LLC, College Station, TX, USA). All data were assessed for normality using the Wilks-Shapiro test and checked for outliers using the IQR rule prior to analysis. Repeated measures analyses used the Greenhouse-Geisser correction for sphericity. *Post-hoc* tests used the Bonferroni correction for multiple comparisons. All values reported are mean ± SEM. For purposes of statistical significance *p* < 0.05 was considered significant. Where the data were found to not be normally distributed and to contain a large number of outliers (>5%) the data were analyzed using the Skillings-Mack test for within-subjects factors and the Kruskal Wallis test for between-subjects factors. All other data were analyzed using analysis of variance (ANOVA). Between-subjects factors included: dose (of IV DA), and drug (pretreatment condition). Within-subjects factors included: time and aCSF type (standard or modified). Each analysis included all relevant factors.

## Results

### IV DA Effects on DA Levels in the NAc

**Figure 1:**
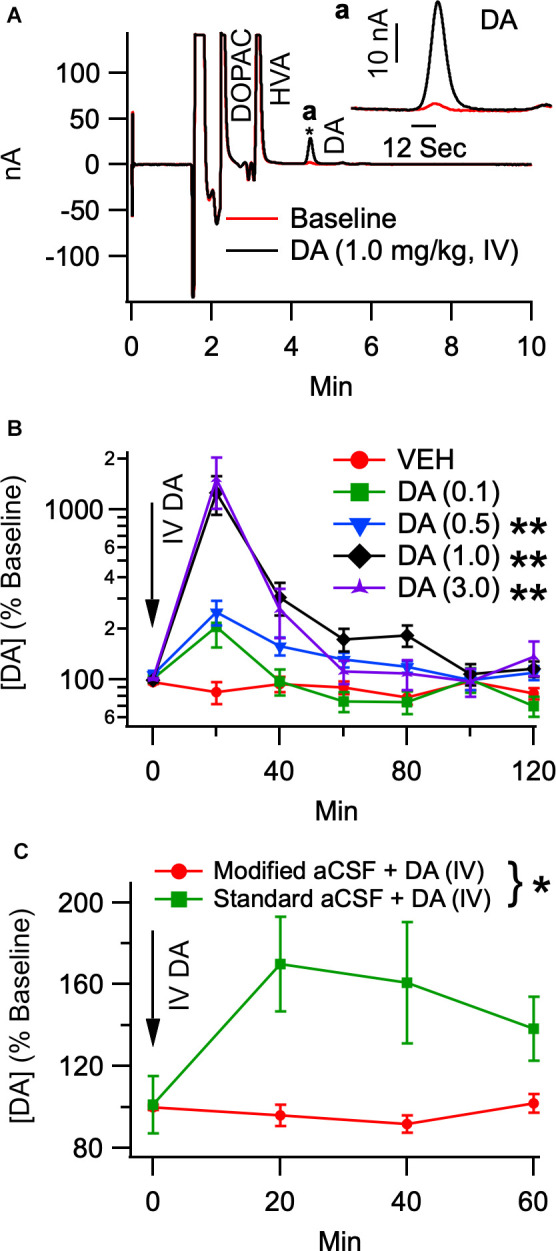
Intravenous DA enhances dopamine release in the NAc in a dose-dependent manner. **(A)** Representative chromatogram of dialysate analyzed by HPLC before and after IV DA (1.0 mg/kg). The trace from a final baseline sample is superimposed with the trace from the first collection following an infusion of IV DA. The inset in the top right corner displays a magnified view of only the DA peaks from the two collections. An asterisk indicates where the inset is zoomed in. **(B)** Dopamine levels in the NAc were dose-dependently enhanced by IV DA doses of 0.1 mg/kg (*n* = 8), 0.5 mg/kg (*n* = 8), 1.0 mg/kg (*n* = 18), and 3.0 mg/kg (*n* = 10). This was most pronounced in the first 20 min after IV DA administration. **(C)** To determine whether IV DA enhanced DA levels in the NAc through an action potential dependent mechanism IV DA (1.0 mg/kg) was administered twice in the same rat. One administration occurred while the probe was being perfused with standard aCSF while another administration occurred while the probe was being perfused with modified aCSF, revealing that IV DA was unable to enhance DA levels in the NAc in the presence of lidocaine and the absence of calcium. Asterisk *, ** indicates *p* < 0.05 and *p* < 0.01, respectively.

To determine the effects of IV DA on DA levels in the NAc, rats received IV injections of DA at the following doses: 0.1 mg/kg (*n* = 8), 0.5 mg/kg (*n* = 8), 1.0 mg/kg (*n* = 18), 3.0 mg/kg (*n* = 10), as well as a vehicle injection (*n* = 8). Figure 1A displays a representative chromatogram showing the effects of IV DA on DA concentrations in dialysate samples taken from the NAc. Data were analyzed using the Kruskal Wallis test to assess differences in DA levels between treatment conditions and the Skillings-Mack test to assess changes in DA levels over time. This analysis revealed that IV DA enhanced DA levels in the NAc in a dose-dependent manner (*H*_(4)_ = 49.75, *p* < 0.01; Figure 1B) with *post-hoc* (Dunn’s test) analysis indicating that 3.0 (*z* = −3.77, *p* < 0.01), 1.0 (*z* = −5.67, *p* < 0.01), and 0.5 mg/kg IV DA (*z* = −4.59, *p* < 0.01) significantly enhanced NAc DA levels. Additionally, DA levels in the NAc were found to vary with time [Skillings-Mack (SM) = 65.40, *p* < 0.01].

To determine whether the increase in DA levels seen in the NAc following the administration of IV DA was due to action potential dependent release, 11 rats received two injections of DA (1.0 mg/kg, IV) separated by a minimum of 5 h. These injections occurred under urethane anesthesia with one occurring while standard aCSF was being perfused through the probe and the other occurring while modified aCSF was being perfused. The time between the two injections was sufficient for a stable baseline to be achieved prior to the second injection. The order of the injection conditions was counterbalanced to account for any order effects. Analysis of the data revealed that modified aCSF greatly attenuated the effect of IV DA (1.0 mg/kg) on DA levels in the NAc (SM = 7.36, *p* = 0.01; Figure 1C) and that DA levels were stable over time (SM = 3.98, *p* = 0.26). The baseline DA concentrations under urethane anesthesia were 2.7 ± 0.9 nM (standard aCSF) and 1.3 ± 0.6 nM (modified aCSF).

### Effects of Blockade of Peripheral D2Rs on DA Levels in the NAc

**Figure 2:**
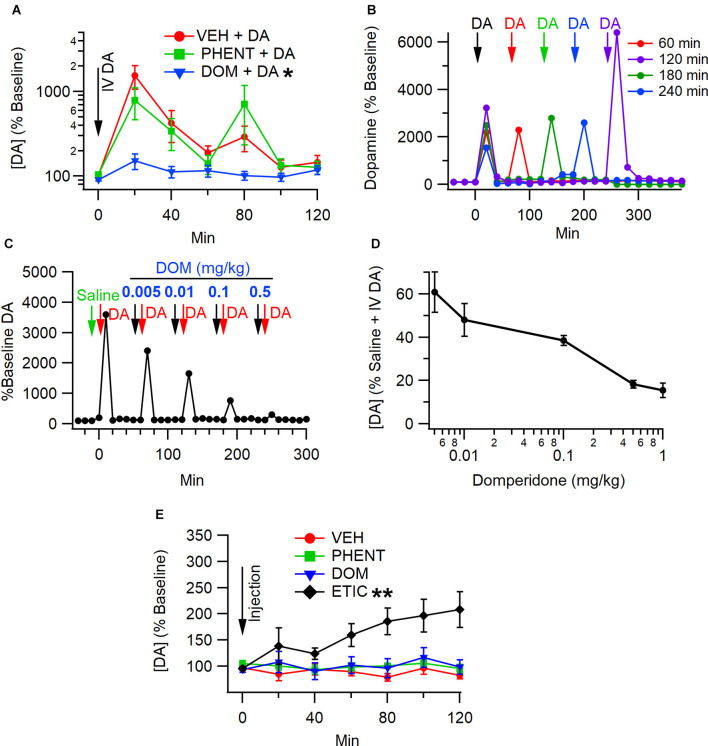
Antagonismof peripheral D2Rs blocks enhancement of NAc DA levels by IV DA. **(A)** IV administration of DA (1 mg/kg) enhanced DA levels in the NAc in the presence of a vehicle (VEH). In the presence of domperidone (DOM), a peripheral D2R antagonist, the effect of IV DA on NAc DA levels was attenuated, while phentolamine (PHENT) had no effect on NAc DA levels following IV DA. Dose-response for domperidone on IV DA enhancement of NAc DA release. **(B)** Interval dependency of sequential IV DA injections (1.0 mg/kg). These are four representative experiments at four intervals (60, 120, 180, and 240) showing that IV enhancement of DA is repeatable at 60 min intervals, but there is some potentiation at long intervals. **(C)** Representative experiment showing the effects of increasing doses of domperidone (DOM) on IV DA (1 mg/kg) enhancement of DA levels in the NAc. **(D)** Domperidone reduces IV DA enhancement of NAc DA release with an IC50 of 0.01 mg/kg. A dose of 1 mg/kg was chosen in most experiments as it suppressed IV DA enhancement of DA release. Summary of domperidone dose-response. **(E)** Vehicle (1 ml/kg, IV), DOM (1 mg/kg, IV), and PHENT (1 mg/kg, IV) did not alter NAc DA levels on their own. Eticlopride (1 mg/kg, IV), a centrally active D2R antagonist, enhanced DA levels in the NAc. * indicates *p* < 0.05, ** indicates *p* < 0.01.

To determine whether antagonism of peripheral D2Rs would block IV DA-mediated enhancement of DA levels in the NAc, rats were pretreated with DOM (1 mg/kg, IV; *n* = 8), PHENT (1 mg/kg, IV; *n* = 8), or VEH (1 ml/kg, IV; *n* = 18) 5 min prior to the administration of IV DA (1 mg/kg). Analysis of the data using the Kruskal Wallis and Skillings-Mack tests revealed that the degree to which IV DA enhanced NAc DA levels was dependent on the pretreatment condition (*H*_(2)_ = 12.48, *p* < 0.01; Figure 2A) and that DA levels in the NAc were found to vary over time following IV DA administration (SM = 57.81, *p* < 0.01). A *post-hoc* analysis using Dunn’s test determined that PHENT did not significantly alter the effect of IV DA on NAc DA levels (*z* = 0.50, *p* = 1.00), while DOM pretreatment significantly reduced the effect of IV DA administration on NAc DA levels relative to pretreatment with VEH (*z* = 3.48, *p* < 0.01) or PHENT (*z* = 2.54, *p* = 0.03).

To determine whether the effects of DOM on IV DA enhancement of NAc DA levels were dose-dependent, escalating doses of DOM (0.005 mg/kg–1.0 mg/kg) separated by 60 min between doses were co-administered with phentolamine (1.0 mg/kg) 10 min prior to treatment with IV DA. Domperidone decreased IV DA enhancement of DA levels in the NAc in a dose-dependent manner (SM = 14.45, *p* = 0.01; Figure 2D). The IC_50_ for DOM was approximately 0.01 mg/kg. Figure 2B shows the interval dependency of repeated IV DA infusions. Figure 2C shows a representative trace of DA release in response to IV DA administration and pretreatment with increasing doses of DOM, showing decreased DA release with larger doses of DOM.

To assess whether PHENT or DOM alone affected DA levels in the NAc rats, they were administered DOM (1 mg/kg, IV; *n* = 8), PHENT (1 mg/kg, IV; *n* = 8), ETIC (1 mg/kg, IV; *n* = 8) or VEH (1 ml/kg, IV; *n* = 8) and microdialysis was performed as previously described. These data were analyzed using the Skillings-Mack test. Analysis of these data indicated that there was an effect of treatment condition on DA levels in the NAc (SM = 28.87, *p* < 0.01; Figure 2E) and that DA levels in the NAc were stable over time (SM = 8.00, *p* = 0.21). The sign test was used to perform pairwise comparisons to determine which treatment conditions altered NAc DA levels. This analysis revealed that only ETIC produced significant increases in NAc DA levels and that it did so relative to VEH (*p* < 0.01), DOM (*p* < 0.01), and PHENT (*p* = 0.01).

### Effects of IV DA on Putative VTA DA Neuron Firing Rate

**Figure 3:**
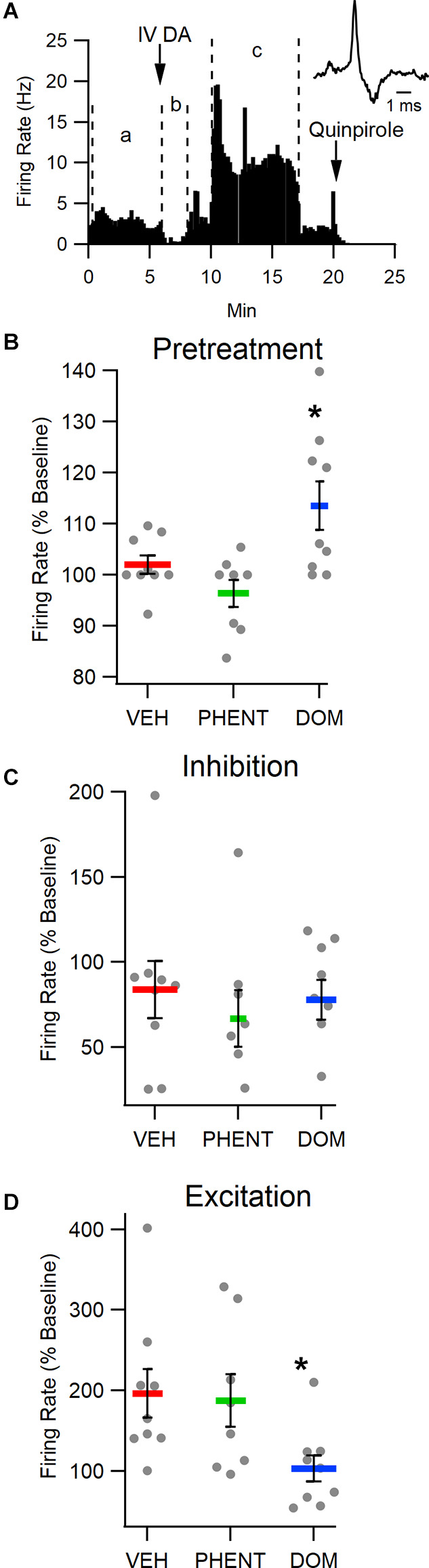
Intravenous DA enhances the firing rate of putative DA neurons in the VTA. **(A)** Representative trace of the firing rate of a putative DA neuron in an experiment in which IV DA was administered. Note the transient, inhibition from baseline (between lines marked in a) of firing rate immediately after the injection (between lines marked b) and the more prolonged increase in firing rate following the transient inhibition (between lines marked c). **(B)** The firing rate of putative DA neurons was enhanced by domperidone (DOM; 1 mg/kg, IV) pretreatment. **(C)** The magnitude of inhibition produced by IV DA (3.0 mg/kg, IV) administration was not affected by pretreatment with vehicle (VEH), phentolamine (PHENT), or DOM. **(D)** However, DOM blocked the enhancement of the DA neuron firing rate produced by IV DA. Phentolamine did not significantly decrease this effect. * indicates *p* < 0.05.

To determine the effect of IV DA on putative VTA DA neuron activity, rats were administered IV DA (3.0 mg/kg) preceded by an injection of one of VEH (*n* = 9), PHENT (*n* = 8), or DOM (*n* = 9). IV DA had a clear, biphasic effect on the firing rate of most putative DA neurons in the VTA. This effect was characterized by a brief inhibition beginning almost immediately after administration of IV DA and lasting for an average of 90 s followed almost immediately by an excitation lasting for an average of 330 s (Figure 3A). The inhibition was present in eight/nine of the recorded neurons in the VEH treatment group with robust excitation also present in 8/9 of the recorded neurons. Each putative DA neuron recorded displayed at a minimum an excitatory or an inhibitory response to IV DA. There was an effect of the pretreatment condition on DA neuron firing (*H*_(2)_ = 7.89, *p* = 0.02; Figure 3B). This effect was driven by a slight increase in DA neuron activity in the first 5 min following pretreatment with DOM, albeit this increase was only significant in relation to the PHENT (*z* = −2.80, *p* = 0.02) and not the VEH (*z* = −1.57, *p* = 0.35) pretreatment group. The inhibition of the DA neuron firing rate produced by IV DA (*z* = 1.37, *p* = 0.51; Figure 3C) was not dependent on the pretreatment condition. The excitation produced by IV DA was affected by the pretreatment condition (*H*_(2)_ = 7.54, *p* = 0.02; Figure 3D). A *post-hoc* analysis using Dunn’s test indicated that DOM reduced VTA DA neuron activation following IV DA relative to the VEH (*z* = 2.56, *p* = 0.03) but not the PHENT (*z* = 2.12, *p* = 0.11) pretreatment condition.

Microdialysis samples were also collected during these experiments (baseline and 0–20 min timepoint only, baseline DA = 3.4 ± 0.4 nM). This data was then compared with the data from the freely moving microdialysis experiments using the Wilcoxon rank-sum test (IV DA 3.0 mg/kg, baseline DA = 7.2 ± 0.8) to determine whether urethane anesthesia affected IV DA mediated DA release. This analysis revealed that there was not a significant effect of urethane anesthesia on IV DA mediated DA release [*z* = −0.74, *p* = 0.46; freely moving (values are % baseline): 1,579 ± 549, *n* = 10; urethane anesthetized: 2,808 ± 856, *n* = 9; *z* = 0.16, *p* = 0.87; freely moving (nM increase in DA concentration): 107.4 ± 38.9, *n* = 10; urethane anesthetized: 92.5 ± 32.6].

### IV DA Place Conditioning

**Figure 4:**
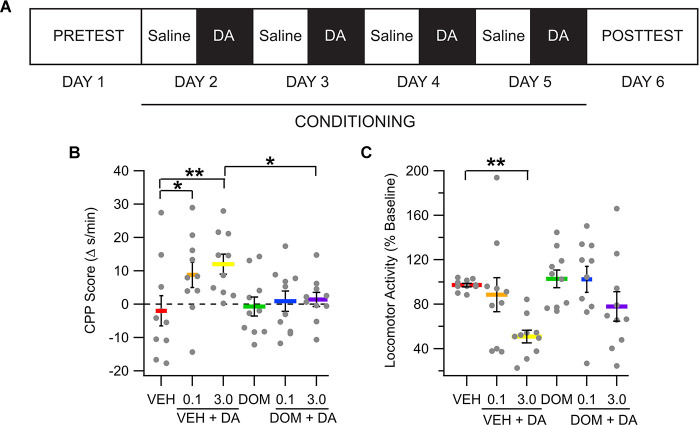
Intravenous DA produces conditioned place preference. **(A)** Diagram showing the timing of different phases of the place conditioning experiments. **(B)** The amount of time spent on the CS+ floor increased in the IV DA (0.1 mg/kg) and IV DA (3.0 mg/kg) groups. This effect was attenuated by pretreatment with domperidone (DOM; 1 mg/kg, IV). **(C)** The IV DA (3.0 mg/kg) group had significantly reduced locomotor activity during CS+ sessions as compared with the vehicle group. * indicates *p* < 0.05, ** indicates *p* < 0.01.

To determine whether IV DA would produce place conditioning, rats received CS+ trials in which VEH (1 ml/kg, IV), DOM (1 mg/kg, IV), VEH + IV DA (0.1 or 3.0 mg/kg, IV), or DOM + IV DA were administered. There were 10 rats in each treatment group. Figure 4A displays the experimental timeline for place conditioning experiments. Data were analyzed using a two-way ANOVA with dose and DOM as between-subjects factors. At pretest there was no statistical difference among the various groups in their initial preference for the CS+ floor (main effect dose: *F*_(2,54)_ = 1.08, *p* = 0.35; main effect DOM: *F*_(1,54)_ = 0.00, *p* = 0.95; dose × DOM interaction: *F*_(2,54)_ = 0.09, *p* = 0.92). Analysis of the difference scores found that rats treated with IV DA increased the time that they spend on the CS+ floor [main effect dose; *F*_(2,54)_ = 3.25, *p* = 0.047, partial *η*^2^ = 0.11 (0.00, 0.25); Figure 4B], however this increase was attenuated by pretreatment with DOM [main effect DOM: *F*_(1,54)_ = 4.51, *p* = 0.04, partial *η*^2^ = 0.08 (0.00,0.23)]. There was not a significant interaction betweenDOM and the dose of DA administered (dose × DOM interaction; *F*_(2,54)_ = 1.77, *p* = 0.18).

Locomotor activity from the pretest and posttest sessions was compared using a mixed model ANOVA with test session as a within-subjects factor and treatment group and dose as between-subjects factors. This analysis revealed no significant differences in locomotor activity on the basis of any of the factors (main effect dose; *F*_(2,54)_ = 0.98, *p* = 0.38; main effect DOM: *F*_(1,54)_ = 0.09, *p* = 0.76; dose × DOM interaction: *F*_(2,54)_ = 0.98, *p* = 0.38; main effect session: *F*_(1,54)_ = 0.43, *p* = 0.51; dose × session interaction: *F*_(2,54)_ = 0.34, *p* = 0.72; DOM × session interaction: *F*_(1,54)_ = 3.11, *p* = 0.08; dose × DOM × session interaction: *F*_(2,54)_ = 1.48, *p* = 0.24; not shown).

Locomotor activity during conditioning trials was compared between groups to determine whether IV DA impacted locomotor activity in rats. All values are expressed as a percentage of locomotor activity during CS− (saline) trials. Data were analyzed using a two-way ANOVA with DOM and dose as between-subjects factors. Analysis of this data indicated that IV DA produced a marked decrease in locomotor activity during conditioning trials [main effect dose: *F*_(2,54)_ = 6.98, *p* < 0.01, partial *η*^2^ = 0.21 (0.03, 0.36); Figure 4C]. There was not a significant effect of DOM on locomotor activity (main effect DOM: *F*_(1,54)_ = 3.35, *p* = 0.07) nor was there an interaction between the dose of IV DA administered and DOM treatment status (dose × DOM interaction: *F*_(2,54)_ = 0.54, *p* = 0.58).

### Effects of IV DA on USVs

**Figure 5:**
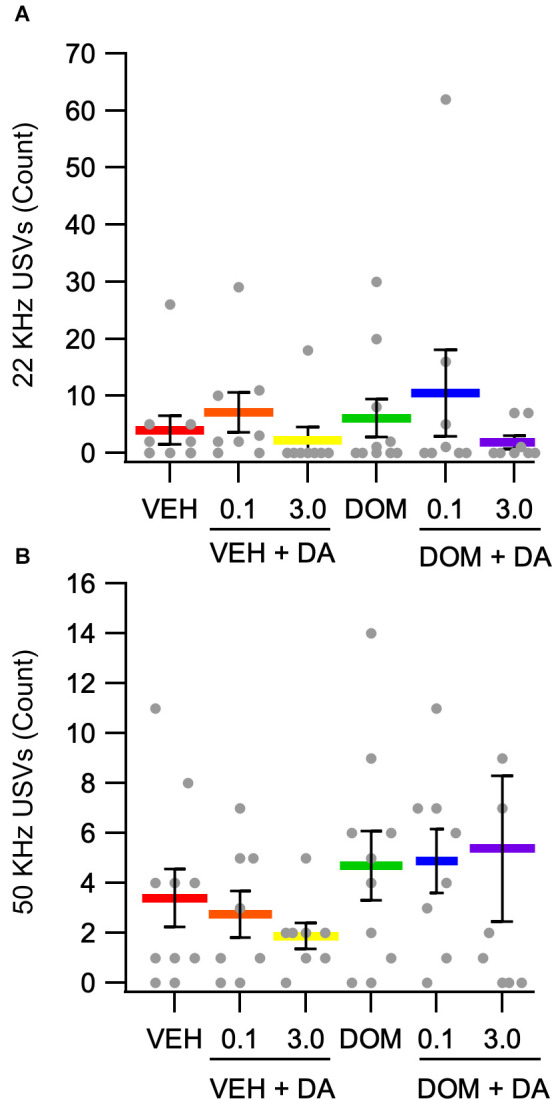
Counts of positive and negative affect associated vocalizations. **(A)** There was no effect of IV DA (0.1, 3.0 mg/kg) or domperidone (DOM; 1.0 mg/kg, IV) on negative affect associated 22 kHz USVs. **(B)** The number of positive affect associated 50 kHz USVs was not significantly altered by treatment condition.

Ultrasonic vocalizations were recorded during conditioning sessions. Due to an equipment malfunction the recordings for eight animals were lost. These animals were equally distributed across the VEH + IV DA [0.1 (*n* = 2), 3.0 (*n* = 2) mg/kg], and DOM + IV DA [0.1 (*n* = 2), 3.0 (*n* = 2) mg/kg] groups. The number of negative affect and positive affect associated calls from conditioning trials were compared across treatment groups using the Kruskal Wallis test. This analysis revealed that there was no effect of dose or DOM on the number of negative (main effect dose: *H*_(2)_ = 4.45, *p* = 0.11; main effect DOM: *H*_(1)_ = 0.00, *p* = 1.00) or positive (main effect dose: *H*_(2)_ = 0.95, *p* = 0.62; main effect DOM: *H*_(1)_ = 1.80, *p* = 0.18) affect related vocalizations (Figures 5A,B).

## Discussion

Previously it has been shown that peripherally administered DA enhances DA levels in the NAc and affects a range of DA-related behaviors. This study examined the effects of IV DA administration on DA levels in the NAc and putative DA neuron activity in the VTA. It also investigated the effects of IV DA in a place conditioning paradigm and measured ultrasonic vocalizations. Intravenous DA enhanced DA levels in the NAc in a dose-dependent manner. It also produced a biphasic effect on most VTA DA neurons recorded, with a transient inhibition occurring shortly after IV DA administration followed by a period of increased activity. Domperidone reduced IV DA enhancement of NAc DA levels in a dose-dependent manner and attenuated the increase in putative DA neuron firing rate following IV DA suggesting a role for D2Rs in mediating these effects. Intravenous DA also increased the time spent on the DA paired side of a place conditioning apparatus and DOM reduced this effect. Ultrasonic vocalizations, however, were not influenced by IV DA administration.

### IV DA Enhancement of DA Levels in the NAc as Measured by Microdialysis

In the present study, IV DA produced dose-dependent increases in DA levels in the NAc. The peak increases ranged from approximately two to 15 times greater than the baseline DA levels depending on the dose of IV DA administered, rendering them similar in magnitude to those seen following the administration of drugs of abuse such as amphetamine (Di Chiara and Imperato, 1988). To test whether these increases were the result of action potential dependent DA release or if they came from another source, such as DA leaking across the BBB, we perfused a modified aCSF containing 37 mM lidocaine and 0 mM Ca^2+^ through the microdialysis probe. While perfusion with this modified aCSF blocked increases in NAc DA levels following IV DA administration, the increase observed during perfusion with the standard aCSF was also reduced relative to earlier experiments. It is unclear why this occurred. One possibility was that urethane anesthesia attenuated the effects of IV DA on NAc DA levels. This does not appear to be the case, however, as an analysis of dialysate samples obtained during the single unit recordings which were also carried out under urethane anesthesia did not display a similar effect. As such, this data is difficult to interpret and does not fully rule out the possibility that the increases in NAc DA levels following IV DA administration result from DA crossing the BBB. The present study extends previous work showing that intranasal DA and IP DA enhance DA levels in the NAc (de Souza Silva et al., 2008). Although the time course of this effect in the present study differs slightly from that previously reported these differences are likely the result of the different routes of administration used.

### IV DA Enhancement of DA Levels in the NAc Can Be Attenuated by DOM

Pretreatment with DOM dose dependently reduced IV DA mediated augmentation of DA levels in the NAc. Domperidone is a peripheral D2R antagonist that does not readily cross the BBB (Laduron and Leysen, 1979; Michiels et al., 1981). Although DOM can be detected at low levels in the brain following large doses (≥2.5 mg/kg, IV) it is principally detected in paraventricular regions (Laduron and Leysen, 1979; Heykants et al., 1981; Michiels et al., 1981). As such, DOM mediated reductions in IV DA driven increases in NAc DA levels likely result, at least in part, from the blockade of receptors outside the BBB. Consistent with this hypothesis is the finding that DOM alone did not enhance NAc DA levels while a centrally active D2R antagonist, ETIC, did.

An important consideration in determining whether DOM affects IV DA enhancement of NAc DA release through D2Rs is the selectivity of DOM for these receptors. Despite being highly selective for D2Rs relative to D1Rs (Kohli et al., 1983; Andersen et al., 1985) and α-2 adrenergic receptors (Kohli et al., 1983), DOM also acts as an α1-adrenergic receptor antagonist (Ennis and Cox, 1980). To control for the possibility that the effects of DOM were mediated by off-target effects two approaches were used. First, in one treatment condition rats received phentolamine prior to receiving IV DA. As phentolamine did not reduce IV DA enhancement of NAc DA release, this indicated that the effects of DOM were not mediated solely by its actions at α1-adrenergic receptors. Second, a dose response curve for the effects of DOM was constructed to determine the amount of DOM required to block the effects of IV DA. The dose response curve for DOM indicated that the IC_50_ was approximately 0.01 mg/kg. A previous report had found the ED_50_ for DOM inhibition of apomorphine-induced emesis to be 0.003 mg/kg (IV) and 0.03 (oral) (Niemegeers et al., 1980). The IC_50_ for DOM in the present study is slightly higher than what was previously reported. This may be related to the dose of agonist used. In the present study the IC_50_ of DOM was calculated against the administration of 1 mg/kg IV DA whereas in the previous study, it was calculated against 0.31 mg/kg sc apomorphine. Considering this, it seems reasonable to conclude that the effects of DOM on IV DA enhancement of NAc DA levels reflect actions at D2Rs, possibly in conjunction with actions at α1-adrenergic receptors.

### IV DA Enhances the Firing Rate of Putative DA Neurons in the VTA

Intravenous DA had a biphasic effect on putative VTA DA neuron activity. This effect was characterized by a brief inhibition of VTA DA neuron activity followed shortly thereafter by increased activation. This finding extends earlier research that had used indirect measures to provide evidence of enhanced DA neuron activity following peripheral DA administration (Pum et al., 2009). In agreement with the microdialysis data, pretreatment with DOM reduced the excitation but not the inhibition of DA neurons following IV DA administration. Phentolamine did not alter either the inhibitory or the excitatory responses of the neurons. The rapid inhibition of VTA DA neurons by IV DA could be taken to indicate that IV DA is crossing the BBB and inhibiting VTA DA neurons by binding to D2Rs however, the duration of this inhibition is brief (90 s on average) and this theory is difficult to justify in light of the subsequent increase in DA neuron firing. Additionally, if DOM and IV DA were exerting their effects by crossing the BBB DOM would be expected to inhibit the initial inhibitory phase of IV DA rather than the subsequent excitatory phase. Thus, these findings suggest that IV DA and DOM are acting at D2Rs outside the BBB.

In assessing the effects of DOM and PHENT alone on DA neuron activity it was found that relative to PHENT DOM produced an increase in the firing rate of VTA DA neurons during the first 5 min following its administration. This effect was relatively modest (113 ± 5% baseline) and was not significant when compared with VEH-treated animals, which may help explain why DOM did not produce a discernible change in NAc DA levels during the microdialysis experiments. That DOM increased the firing rate of VTA DA neurons could indicate that a small but physiologically relevant amount of the drug was able to pass the BBB during these experiments as acute treatment with D2R antagonists is known to enhance VTA DA neuron activity (Bunney and Grace, 1978).

### IV DA Produces Conditioned Place Preference in a Place Conditioning Paradigm

In a place conditioning paradigm, it was found that both a low dose (0.1 mg/kg) and a high dose (3.0 mg/kg) of IV DA enhanced preference for the DA paired chamber. This effect was blocked by pretreatment with DOM on the conditioning days. One interpretation of this finding is that IV DA is rewarding, and that DOM blocks the reinforcing effects of IV DA. An alternative explanation, given the biased design employed in this study, is that IV DA is anxiolytic and thus increases the time spent in the IV DA paired chamber because its anxiolytic properties overcame the initial aversion to the less preferred chamber. The average time spent in the less preferred chamber on the pretest day by the VEH + IV DA treated rats (combined 0.1 and 3.0 mg/kg) was 21 ± 1 s/min, and the average time spent in the same chamber on the posttest day was 31 ± 3 s/min, which is to say that rather than demonstrating an absolute preference for the CS+ paired chamber on the posttest day the rats demonstrated an increased preference for the CS+ paired chamber relative to the pretest day. This could also indicate that the increased preference for the IV DA paired chamber may reflect the anxiolytic properties of IV DA (Tzschentke, 1998). Locomotor activity did not significantly differ between the pretest and posttest sessions indicating that the change in preference for the CS+ chamber was not artifactual to a change in locomotor activity.

To better contextualize the observed effects of IV DA in a place conditioning paradigm it may be helpful to compare them to those produced by drugs of abuse such as amphetamine and cocaine which also enhance DA levels in the NAc. Although by no means a comprehensive review, we identified several cocaine and amphetamine conditioned place preference studies that used a biased apparatus and paired the drug with the less preferred chamber as was done in the present study. In these studies, cocaine increased the time spent in the CS+ chamber by an average of approximately 15 s/min (posttest − pretest: 7 s/min low and 27 s/min high), while failing to produce an absolute preference for the CS+ chamber, at least based on the data available in the manuscripts (average time in CS+ chamber: 30 s/min, 27 s/min low and 33 s/min high; Bardo et al., 1986; Spyraki et al., 1987; Yates et al., 2021; Nedelescu et al., 2022; Philogene-Khalid et al., 2022). Amphetamine similarly increased the time spent in the CS+ chamber (posttest − pretest = 17 s, low = 13 s, high = 23 s) however, it was not possible to determine whether amphetamine produced an absolute or relative preference in these studies (Spyraki et al., 1982; Gilbert and Cooper, 1983; Decker et al., 2018). Intravenous DA enhanced the time spent in the CS+ chamber by 9 s (0.1 mg/kg) and 12 s (3.0 mg/kg), while failing to produce an absolute preference for the DA paired chambers. The increase in preference for the IV DA paired chamber was relatively similar to that seen following cocaine place conditioning and somewhat smaller than that observed following amphetamine place conditioning. Although caution should be exercised in the conclusions drawn given that these studies only represent a small subset of all cocaine and amphetamine place conditioning studies and given that there are several differences in the experimental procedures between the studies, it suggests that the observed effects of IV DA on place conditioning are not altogether inconsistent with those observed with drugs of abuse such as cocaine and amphetamine.

Intravenous DA reduced locomotor activity during conditioning trials. This effect was not altered by pretreatment with DOM. This finding contrasted with the expected outcome that locomotor activity would be enhanced by IV DA. Previous studies in which peripheral DA was administered had mixed results with regard to the effects of IV DA on locomotor activity with one study showing increased locomotor activity 20–30 min after IN DA administration (de Souza Silva et al., 2008) and another showing no effect (Buddenberg et al., 2008). Based on these results it seems likely that the route of administration, as well as the duration and timing of the trial after peripheral DA administration, may determine whether peripherally administered DA produces an increase, decrease, or no effect on locomotor activity.

### Effects of Peripheral D2Rs on USVs

In this study, IV DA did not alter the number of positive or negative affect associated USVs emitted over the course of four conditioning sessions. There was also no effect of DOM on the number of vocalizations emitted. Overall, the number of calls emitted by rats was low with only 2.3 ± 0.2 calls emitted per session on average. Further, seven rats did not emit any detectable vocalizations across all conditioning sessions. An additional 21 rats did not emit any detectable 22 KHz calls while four additional rats did not emit any detectable 50 KHz calls. Previous work has shown that intranasal DA decreased 22 KHz calls in rats during restraint stress (Talbot et al., 2017) demonstrating that peripherally administered DA can modulate USV production in response to external stimuli. Although peripherally administered DA can affect USV production in response to external stimuli this study indicates that peripherally administered DA on its own is not a strong driver of USV production. As such, the effects of IV DA on vocalizations may be limited to, or at least best studied in, settings where robust vocalization is expected.

### Possible Mechanisms and Limitations

The purpose of this study was to assess the role of peripheral D2R activation in IV DA enhancement of DA levels in the NAc. After careful consideration of the findings in this study, this question remains to some degree unanswered. While we confirmed previous findings that peripherally administered DA enhances DA levels in the NAc (de Souza Silva et al., 2008) we did not conclusively show that the ability of DOM to attenuate this effect is due solely to the activation of peripheral D2Rs. The finding that DOM but not PHENT attenuated IV DA enhancement of DA levels in the NAc is promising, as is an IC_50_ of 0.01 mg/kg for the effect of DOM, but neither finding conclusively demonstrates that the effect of DOM is solely related to the actions of DOM at D2Rs and not related to the blockade of both D2Rs and α-adrenergic receptors. This same critique holds for the single-unit data showing that DOM attenuated the activation of putative DA neurons following IV DA administration. In addition, the slight increase in DA neuron activity following DOM administration raises questions as to whether a physiologically relevant concentration of DOM may have been able to cross the BBB and antagonize central D2Rs during the single-unit recordings. Despite these limitations, this study contributes to our understanding of the effects of peripheral DA. This study demonstrated that peripherally administered DA produces a biphasic response in VTA DA neurons and that IV DA has anxiolytic and/or rewarding properties. Finally, it demonstrated that these effects are blocked by pretreatment with DOM.

An additional caveat relates to the interpretation of the dose response for DOM. In the experiment determining the dose-dependency of the effects of DOM, each rat received six doses of IV DA separated by 60 min. While we had previously performed experiments in which two sequential doses of IV DA were given at 60 min intervals without DOM, we did not perform any experiments in which six sequential doses of IV DA were given in the absence of DOM. When two sequential doses of IV DA were given at 60-min intervals in the absence of DOM we did not observe desensitization of the response to IV DA. Despite this, it is possible that with additional doses of IV DA some desensitization occurs. As such, it is possible that a portion of the reduction in response to IV DA attributed to escalating doses of DOM over the course of the third through sixth injections of IV DA could reflect desensitization of the response to IV DA. We do not anticipate that this was the case but in the absence of data showing that there was no desensitization between the third and sixth doses this possibility cannot be entirely ruled out. It is also relevant to note that in subsequent experiments a dose of 1 mg/kg DOM was administered prior to a single infusion of IV DA. In these experiments 1 mg/kg DOM produced stronger inhibition of IV DA enhancement of NAc DA release than was observed following treatment with 0.005 mg/kg DOM. This dose of DOM was given prior to the second infusion of IV DA, which in the absence of DOM we had found to elicit a similar increase in NAc DA levels to the increase observed following the first infusion. This supports the conclusion that although some desensitization of the response to IV DA could have occurred with repeated infusions, the effects of DOM on IV DA enhancement of NAc DA levels were dose-dependent.

Intravenous DA affects blood pressure and vascular perfusion. The nature of these effects is dependent on both dose and infusion rate. At low doses (<3 μg/kg/min), infusions of IV DA act through D1Rs and D2Rs with the net effect of this action being vasodilation (Brodde, 1982; Cavero et al., 1982; Ensinger et al., 1985). At higher doses (>10 μg/kg/min), the primary action of DA is to stimulate vasoconstriction through activation of α1-adrenergic receptors, with intermediate doses producing effects largely mediated by β-adrenergic receptors (Goldberg, 1972). At infusion rates such as those used in this study IV DA increases blood pressure and decreases cerebral blood flow (von Essen et al., 1980). Pretreatment with phentolamine or DOM would be expected to reduce the vasopressor effect of the high dose of IV DA through a blockade of α1-adrenergic receptors.

Although the exact mechanism whereby IV DA modulates VTA DA neuron activity remains unclear the evidence presented suggests that it does so at least in part through a peripheral D2R related mechanism. Previous research has shown that the VTA receives input from regions involved in the processing of interoceptive signals including the arcuate nucleus and the NTS (Geerling and Loewy, 2006; Alhadeff et al., 2012; Metz et al., 2021). Both these regions are part of zones where the permeability of the BBB is increased allowing for easier trafficking of molecules across the BBB. As neuronal populations in both the NTS and the arcuate nucleus express D2Rs (Lawrence et al., 1995; Hyde et al., 1996; Yoon and Baik, 2015; Romanova et al., 2018) and would be infiltrated by IV DA, these regions may be involved in mediating the peripheral D2R related effects of IV DA on VTA DA neurons. Future research could explore these possibilities.

It is also noteworthy that a previous research study explored the effects of changes in blood pressure on the activity of VTA neurons (Kirouac and Ciriello, 1997). In this study, it was shown that some VTA neurons respond with excitation and others with inhibition to changes in blood pressure, and that these changes did not occur in barodenervated animals. While in the present study PHENT did not significantly alter the effect of IV DA on DA neurons, it is possible that the effects of blood pressure on VTA neuron activity were masked by other effects related to the administration of IV DA.

## Conclusions

IV DA enhances DA levels in the NAc in a dose-dependent manner and produces a biphasic response in VTA DA neurons. These effects can be attenuated by DOM, a peripheral D2R antagonist, demonstrating a role for peripheral D2Rs in mediating these effects. This study also used a place conditioning paradigm to demonstrate that IV DA possesses anxiolytic and/or rewarding properties. By better understanding the pathways underlying the effects of peripherally administered DA novel targets for modulating central DA function may be found.

## Data Availability Statement

The raw data supporting the conclusions of this article will be made available by the authors, without undue reservation.

## Ethics Statement

The animal study was reviewed and approved by Institutional Animal Care and Use Committee at Brigham Young University and Daegu Haany University.

## Author Contributions

JO, CS, EJ, JY, CY, JL, and SS contributed to the design of the experiments. JO, CS, EB, EJ, and JL helped with the data collection. JO, CS, and SS helped with the data analysis. JO, CS, JY, and SS helped prepare the manuscript. All authors contributed to the article and approved the submitted version.

## Funding

This work was supported by the National Institutes of Health (National Institute on Drug Abuse) grant numbers AA020919 and DA035958 to SS.

## Conflict of Interest

The authors declare that the research was conducted in the absence of any commercial or financial relationships that could be construed as a potential conflict of interest.

## Publisher’s Note

All claims expressed in this article are solely those of the authors and do not necessarily represent those of their affiliated organizations, or those of the publisher, the editors and the reviewers. Any product that may be evaluated in this article, or claim that may be made by its manufacturer, is not guaranteed or endorsed by the publisher.

## ABBREVIATIONS

aCSF: Artificial Cerebral Spinal Fluid
BBB: Blood Brain Barrier
DOM: Domperidone
DA: Dopamine
D2R: Dopamine 2-like receptor
D1R: Dopamine 1-like receptor
ETIC: Eticlopride
IP: Intraperitoneal
IV: Intravenous
NAc: Nucleus Accumbens
NTS: Nucleus of the solitary tract
PHENT: Phentolamine
SN: Substancia Nigra
USV: Ultrasonic Vocalization
VEH: Vehicle
VTA: Ventral Tegmental Area.
